# Host-Dependent Competitive Dynamics Between Two *Trichogramma* Parasitoids in Host Eggs

**DOI:** 10.3390/insects17101077

**Published:** 2026-10-18

**Authors:** Yu Wang, Zhi-Da Li, Jing-Yi Sun, Atif Idrees, Chen Zhang

**Affiliations:** 1 Agricultural College, Jilin Agricultural Science and Technology College, Jilin 132101, China; wangyu@jlnku.edu.cn (Y.W.); lzz1023080787@gmail.com (Z.-D.L.); 2 College of Plant Protection, Jilin Agricultural University, Changchun 130118, China; 16604447236@163.com; 3 Guizhou Provincial Key Laboratory for Agricultural Pest Management of the Mountainous Region, Scientific Observing and Experimental Station of Crop Pest in Guiyang, Ministry of Agriculture and Rural Affairs, Institute of Entomology, Guizhou University, Guiyang 550025, China; atifentomologist@gmail.com

**Keywords:** *Trichogramma dendrolimi*, *Trichogramma ostriniae*, parasitoid competition, host-mediated interaction, molecular detection, biological control, developmental dynamics

## Abstract

Interspecific competition among parasitoids significantly influences the efficiency and stability of biological control programs; however, competitive interactions occurring inside host eggs remain poorly understood because conventional approaches mainly rely on adult emergence outcomes. This study investigated the temporal dynamics of competition between *Trichogramma dendrolimi* and *Trichogramma ostriniae* in two host systems, the eggs of *Ostrinia furnacalis*, the Asian corn borer, and *Corcyra cephalonica*, the rice moth, using species-specific molecular detection. Sequential parasitization assays were performed with reversed introduction sequences (*T. dendrolimi*–*T. ostriniae* and *T. ostriniae*–*T. dendrolimi*), followed by molecular identification of parasitoid persistence at eight developmental time points after parasitization. Three detection states were identified: *T. dendrolimi*-only, *T. ostriniae*-only, and simultaneous *T. dendrolimi* + *T. ostriniae* detection. Results demonstrated that developmental progression was the strongest determinant of parasitoid detection transitions. Oviposition sequence showed no significant overall effect, although descriptive differences between introduction sequences were observed within individual host species. Early developmental stages were characterized by simultaneous detection of both parasitoids, indicating initial coexistence within host eggs. However, simultaneous detection rapidly declined, followed by host-specific dominance patterns. In *O. furnacalis* eggs, *T. ostriniae* became the predominant molecular signal during later development, exceeding 75% detection frequency, whereas *T. dendrolimi* remained comparatively lower. Conversely, in *C. cephalonica* eggs, *T. dendrolimi* showed greater molecular persistence, reaching approximately 60% detection frequency during later developmental stages. Host species significantly influenced molecular detection outcomes, demonstrating host-associated differences in parasitoid molecular persistence. These findings reveal that parasitoid competition is a dynamic developmental process rather than a simple outcome of emergence success and highlight the importance of host-dependent interactions in optimizing *Trichogramma*-based biological control strategies.

## Simple Summary

This study investigated the temporal dynamics of interspecific competition between *Trichogramma dendrolimi* and *Trichogramma ostriniae* in eggs of *Ostrinia furnacalis*, the Asian corn borer, and *Corcyra cephalonica*, the rice moth. Sequential parasitization experiments combined with species-specific molecular detection revealed that parasitoid persistence was strongly associated with host egg developmental progression. The oviposition sequence of the two parasitoid species showed no significant overall effect, although descriptive differences between introduction sequences were observed within individual host species. Both parasitoids initially coexisted within host eggs, followed by host-dependent competitive shifts during later development. *T. ostriniae* showed greater persistence in *O. furnacalis* eggs, whereas *T. dendrolimi* exhibited higher persistence in *C. cephalonica* eggs. These findings highlight that parasitoid competition is a dynamic, host-mediated process and provide valuable insights for optimizing *Trichogramma*-based biological control strategies.

## 1. Introduction

Egg parasitoids belonging to the genus *Trichogramma* Westwood (Hymenoptera: Trichogrammatidae) represent one of the most widely utilized groups of biological control agents worldwide because of their ability to suppress insect pests during the egg stage, thereby preventing larval damage before crop injury occurs [1,2,3]. These minute parasitoid wasps are generally less than 1 mm in body length, a characteristic that enables them to develop within the eggs of numerous insect hosts [4]. Their widespread use in biological control is supported by their ability to parasitize the eggs of numerous economically important lepidopteran pests and by their suitability for mass rearing and augmentative release programs [4].

More than 230 *Trichogramma* species have been described, and several species have been successfully incorporated into augmentative biological control programs against economically important lepidopteran pests [5,6]. Their effectiveness is associated with rapid reproductive capacity, efficient host-searching ability, and adaptability to diverse agricultural environments [7,8,9]. Among these parasitoids, *Trichogramma dendrolimi* Matsumura, 1926 and *Trichogramma ostriniae* Pang & Chen, 1974 are important biological control agents used against several lepidopteran pests, particularly *Ostrinia furnacalis* (Guenée, 1854) (Lepidoptera: Crambidae), the Asian corn borer, one of the most destructive maize pests in Asia [2,10,11,12]. Recent long-term field and laboratory evidence further demonstrated the effectiveness of *T. ostriniae* against *O. furnacalis*, supporting its potential as a sustainable biological control agent in maize production [13]. Recent laboratory investigations have also evaluated the host range of *T. dendrolimi* across different lepidopteran hosts, providing further evidence relevant to its potential application in biological control programs [14]. Beyond *O. furnacalis*, *T. dendrolimi* can successfully parasitize eggs of a diverse range of lepidopteran hosts, including *Dendrolimus sibiricus*, *Dendrolimus pini*, *Macrothylacia rubi*, and *Manduca sexta*, highlighting its relatively broad host range [14]. Other *Trichogramma* species are also associated with economically important lepidopteran hosts; for example, *T. japonicum* has been evaluated against the yellow stem borer *Scirpophaga incertulas* [15]. In addition to natural pest hosts, *Corcyra cephalonica* is widely used as a factitious host for laboratory and mass rearing of *Trichogramma* because it can be maintained continuously under laboratory conditions and provides a readily available source of host eggs [16]. In the present study, *C. cephalonica* was therefore included as a standardized factitious-host system to compare parasitoid persistence with that observed in the target pest host, *O. furnacalis*.

Geographically, *T. dendrolimi* is widely distributed across Eurasia, occurring in numerous European and Asian countries, including China, Japan, Korea, Russia, India, and Kazakhstan, whereas *T. ostriniae* is native to Asia and is particularly associated with maize-growing regions of China [14,17]. The principal host, *O. furnacalis*, is widely distributed across Asia, particularly East and Southeast Asia, whereas *C. cephalonica* is a widely distributed stored-product pest occurring predominantly in tropical and subtropical regions worldwide [18,19]. Both *Trichogramma* species exhibit high parasitization efficiency and have been extensively studied for their potential application in integrated pest management programs. However, when multiple parasitoid species utilize the same host resource, their ecological interactions may strongly influence population establishment, reproductive success, and overall biological control performance [8,10,20,21].

Interspecific competition among parasitoids is a major ecological process that determines species coexistence and competitive dominance within shared hosts [22,23]. Parasitoids competing for the same host may interact through several mechanisms, including host discrimination, oviposition interference, physiological suppression, resource depletion, and differential developmental capacity, which can determine offspring survival, competitive dominance, and biological control outcomes [10,24,25]. Previous studies have demonstrated that competition among egg parasitoids can occur both before and after oviposition, with the final outcome depending on parasitoid species characteristics, host quality, and environmental conditions [10,26,27]. More broadly, closely related parasitoids sharing the same host may coexist through temporal, spatial, behavioral, or other forms of niche partitioning, indicating that competitive interactions should be interpreted within the broader ecological context of resource use and niche differentiation [28].

Sequential parasitization provides an important framework for understanding competitive interactions because the first and second parasitoid attacking the same host may experience different developmental advantages [10,25,27]. Traditionally, the first ovipositing parasitoid was considered to have a priority advantage due to earlier access to host resources and possible physiological manipulation of the host environment [25,29]. However, substantial evidence indicates that competitive outcomes are not always determined by introduction order, and intrinsic competitive ability, immature survival, and host suitability may override simple priority effects [30,31,32,33,34,35]. Recent studies on *Trichogramma* species have similarly demonstrated that oviposition sequence alone cannot fully explain differences in offspring emergence and competitive success, emphasizing the importance of host context and species-specific traits [10].

Molecular diagnostic techniques have recently provided powerful tools for investigating parasitoid–host interactions by allowing direct identification of parasitoid species during immature stages [36,37]. Species-specific PCR markers [36,37], mitochondrial DNA analysis [38,39], and ITS2-based molecular approaches [40,41] have enabled accurate detection of parasitoids within host eggs shortly after parasitization, overcoming limitations associated with emergence-based evaluations. For example, PCR-based detection methods have successfully identified immature *Trichogramma* individuals inside host eggs and provided reliable estimates of parasitism events before adult emergence [38]. Recent advances in molecular quantification have further improved understanding of competitive relationships among parasitoids. Molecular assays based on species-specific markers have been used to quantify parasitism levels and evaluate competitive superiority among egg parasitoid species, demonstrating that molecular approaches can reveal biological interactions that remain undetected through traditional monitoring methods [42].

Despite increasing knowledge regarding parasitoid competition, the temporal dynamics of competitive transitions inside host eggs remain poorly understood. Particularly, little information exists regarding how *T. dendrolimi* and *T. ostriniae* interact during different developmental stages within the same host egg. Determining whether competition is immediately resolved after parasitization or gradually changes during parasitoid development is essential for understanding the ecological mechanisms underlying biological control efficiency. The objectives of this study were to (i) characterize temporal changes in the molecular detection of *T. dendrolimi* and *T. ostriniae* during immature development within host eggs; (ii) determine whether parasitoid introduction sequence influences molecular detection outcomes; and (iii) assess whether these competitive patterns differ between *O. furnacalis* and *C. cephalonica* host eggs. Sequential parasitization experiments with eggs of *O. furnacalis* and *Corcyra cephalonica* (Stainton, 1866) (Lepidoptera: Pyralidae), the rice moth, were conducted to evaluate the effects of host environment and introduction sequence on competitive outcomes. Parasitoid persistence was tracked through *T. dendrolimi*-only, *T. ostriniae*-only, and simultaneous *T. dendrolimi* + *T. ostriniae* detection profiles during immature development. 

## 2. Materials and Methods

### 2.1. Parasitoid Colonies

One laboratory colony of each parasitoid species, *T. dendrolimi* and *T. ostriniae*, was used in this study to investigate the temporal dynamics of interspecific interactions within host eggs. Both parasitoid species were originally collected from parasitized *O. furnacalis* eggs and were maintained under laboratory conditions before experimentation. Prior to the experiments, both parasitoid colonies were maintained on *C. cephalonica* eggs for multiple generations under identical laboratory conditions. *C. cephalonica* was used as a common factitious host to provide a standardized rearing environment for both parasitoid species before experimentation. This common rearing regime reduced variation arising from different immediate rearing conditions; however, potential species-specific effects associated with prolonged rearing on this host cannot be excluded. Parasitoids were maintained in an environmental chamber (Faithful Instrument Co., Ltd., Huanghua, China) at 26 ± 1 °C, 65 ± 5% relative humidity, and a 14:10 h light:dark photoperiod. Newly emerged (<12 h old), mated females were selected for experimental treatments to ensure comparable reproductive status among individuals.

### 2.2. Hosts

To collect experimental eggs, *O. furnacalis* pupae were housed in Bugdorm-I cages (35 × 35 × 35 cm; MegaView Science Co., Ltd., Taichung, Taiwan) under controlled conditions (26 ± 1 °C, 65 ± 5% RH, and a 14:10 h L:D photoperiod). Emerged moths were fed a 10% honey solution, and wax paper strips were suspended inside the cages for oviposition. The resulting egg masses were cut and transferred to a climate chamber (Faithful Instrument Co., Ltd., Huanghua, China) under identical environmental conditions until reaching the required experimental age. The two host species were maintained using different starting stages because they were handled according to their respective laboratory-rearing procedures. For *O. furnacalis*, available pupae were transferred directly to adult emergence and oviposition cages to obtain freshly laid egg masses, whereas *C. cephalonica* was maintained from the larval stage on an artificial diet through pupation and adult emergence to provide a continuous supply of experimental eggs. Concurrently, *C. cephalonica* larvae were reared in plastic containers on a corn-based artificial diet until pupation [43]. Upon emergence, adults were moved to mesh cages (35 × 35 × 35 cm; MegaView Science Co., Ltd., Taichung, Taiwan) positioned over collecting trays. Eggs laid on the cage walls were gently brushed into the trays, filtered through a 0.5 mm mesh to remove debris and scales, and stored under the same optimal conditions (26 ± 1 °C, 65 ± 5% RH, 14:10 h L:D) until use. For *C. cephalonica*, an additional sieving step was necessary because the collected eggs were loose and intermixed with adult scales and other debris. In contrast, *O. furnacalis* deposited cohesive egg masses directly onto the wax paper, allowing the egg masses to be excised and transferred without additional sieving. Utilizing these two distinct host systems allowed for evaluating whether competitive persistence between parasitoid species remains consistent across different host environments or if host-specific characteristics alter developmental trajectories.

### 2.3. Sequential Parasitization Assay

To evaluate interspecific interactions between *T. dendrolimi* and *T. ostriniae*, individual host eggs (0–4 h old) were sequentially exposed to one female of each parasitoid species. In the *T. dendrolimi*–*T. ostriniae* treatment, a single newly emerged, mated *T. dendrolimi* female (<12 h old) was introduced into a glass tube (3 cm in diameter and 10 cm in length; Zhejiang Runlab Technology Co., Ltd., Taizhou, China) containing one host egg and allowed to parasitize it for 10 min. After the *T. dendrolimi* female was removed, a single *T. ostriniae* female was immediately introduced into the same tube and allowed to parasitize the same host egg for an additional 10 min. During each 10-min exposure period, the female was observed continuously, and an oviposition event was recorded when ovipositor insertion into the host egg was observed. Only host eggs for which ovipositor insertion by both parasitoid females was observed were retained for subsequent analysis. The number of parasitoid eggs deposited during each oviposition event was not directly determined; therefore, the procedure confirmed oviposition events rather than the deposition of exactly one parasitoid egg per female. The *T. ostriniae*–*T. dendrolimi* treatment followed the same procedure, except that the order of parasitoid introduction was reversed. Thus, the onset-to-onset interval between the first and second parasitoid exposures was 10 min, with no additional waiting period between exposures. Following the second parasitization event, the eggs were maintained under controlled conditions comprising a 14:10 h light:dark photoperiod, 26 ± 1 °C, and 65 ± 5% relative humidity until sampling. Parasitoid development was monitored at eight developmental time points: 1, 2, 3, 4, 5, 6, 7, and 8 days after the initial parasitization event. These time points represented parasitoid developmental time after initial parasitization and not different intervals between the two parasitoid introductions. At each parasitoid developmental time point, parasitized eggs were collected and stored at −80 °C in an ultra-low-temperature freezer (Qingdao Haier Biomedical Co., Ltd., Qingdao, China) for subsequent DNA extraction and species-specific molecular detection. Each treatment comprised 20 biological replicates. Accordingly, a total of 640 host eggs were analyzed, comprising 320 *O. furnacalis* eggs and 320 *C. cephalonica* eggs. For each host species, 20 biological replicates were included for each parasitoid introduction sequence at each of the eight developmental sampling time points. This time-resolved experimental design allowed the detection of changes in parasitoid coexistence and species-specific persistence before adult emergence, providing information beyond conventional emergence-based assessments.

#### DNA Extraction and Species-Specific Molecular Detection

Genomic DNA was extracted from parasitized host eggs using a CTAB-based extraction method. Individual parasitized eggs were homogenized in 100 μL CTAB extraction buffer containing 2% (*w*/*v*) CTAB, 100 mM Tris-HCl (pH 8.0), 20 mM EDTA, and 1.4 M NaCl, supplemented with 20 μL proteinase K (20 mg/mL). The samples were incubated at 65 °C for 30–60 min to facilitate tissue digestion. After incubation, 120 μL chloroform:isoamyl alcohol (24:1, *v*/*v*) was added, followed by gentle mixing and centrifugation. The aqueous phase was transferred to a new tube, and DNA was precipitated using 100 μL cold isopropanol. The DNA pellet was washed with 500 μL 70% ethanol, air-dried, and dissolved in 30 μL TE buffer. DNA concentration and purity were evaluated using a ScanDrop2 spectrophotometer (Analytik Jena GmbH, Jena, Germany), and extracted DNA samples were stored at −20 °C until PCR amplification [44]. Species-specific molecular markers were used to distinguish *T. dendrolimi* and *T. ostriniae* DNA signals within individual host eggs. PCR amplification was conducted using diagnostic primers previously validated for species discrimination in an A300 Fast Gradient Thermal Cycler (Hangzhou LongGene Scientific Instruments Co., Ltd., Hangzhou, China). Amplified products were separated through polyacrylamide gel electrophoresis and visualized according to standard staining procedures. Based on species-specific amplification patterns, each host egg was assigned to one of three molecular detection categories: *T. dendrolimi*-only detection, *T. ostriniae*-only detection, or simultaneous *T. dendrolimi* + *T. ostriniae* detection. *T. dendrolimi*-only samples contained only the diagnostic band of *T. dendrolimi*, *T. ostriniae*-only samples contained only the diagnostic band of *T. ostriniae*, whereas *T. dendrolimi* + *T. ostriniae* samples showed both species-specific bands within the same host egg.

### 2.4. Statistical Analysis and Modeling of Molecular Detection Patterns

The molecular detection data were analyzed according to host species, oviposition sequence, and developmental time. Each host egg was considered an experimental unit, and detection outcomes were classified into three categorical states: *T. dendrolimi* only, *T. ostriniae* only, and simultaneous detection of both parasitoid species (*T. dendrolimi* + *T. ostriniae*). Detection frequencies were calculated as the proportion of eggs belonging to each molecular detection category within each experimental group.

To evaluate the effects of host species, developmental time, and oviposition sequence on parasitoid detection outcomes, a multinomial generalized linear model (GLM) with a multinomial probability distribution and a generalized logit link function was fitted because the response variable consisted of three mutually exclusive categorical outcomes. Developmental time was treated as a categorical predictor with eight levels (days 1–8) to allow detection probabilities to vary across sampling days without imposing a linear temporal relationship. This approach was selected because the primary objective was to characterize stage-specific changes in molecular detection patterns rather than to estimate a single linear trend over time. The initial multinomial model included the main effects of host species, developmental time, and oviposition sequence, together with all two-way interactions (host × developmental time, host × oviposition sequence, and developmental time × oviposition sequence). The contribution of the interaction terms was evaluated by comparing the model containing all two-way interactions with the main-effects model using a likelihood-ratio test.

Pearson’s chi-square tests were additionally performed to examine overall associations between molecular detection categories and the experimental factors, and Cramer’s V was calculated as a measure of effect size. Temporal changes in detection patterns were evaluated by comparing the relative frequencies of the *T. dendrolimi*-only, *T. ostriniae*-only, and *T. dendrolimi* + *T. ostriniae* detection categories across developmental days (1–8 days after parasitization). Where applicable, post hoc comparisons were used to identify significant differences among groups, with different lowercase letters indicating statistically significant differences. All statistical analyses were performed using IBM SPSS Statistics version 29.0 (IBM Corp., Armonk, NY, USA). Statistical significance was determined at *p* < 0.05. Graphical representations were generated using GraphPad Prism version 10.0 (GraphPad Software, San Diego, CA, USA).

## 3. Results

A total of 640 host eggs were analyzed, comprising two host species (*Ostrinia furnacalis* and *Corcyra cephalonica*), two parasitoid introduction sequences, and eight developmental sampling points. Molecular detection outcomes were classified into three categories: *T. dendrolimi* only, *T. ostriniae* only, and simultaneous detection of both species.

The initial multinomial GLM included host species, developmental time, and oviposition sequence, together with their two-way interactions. The inclusion of interaction terms did not significantly improve model fit (likelihood-ratio χ^2^ = 5.40, df = 30, *p* > 0.999). Consequently, the main-effects model was retained, revealing significant effects of developmental time (χ^2^ = 469.02, df = 14, *p* < 0.001) and host species (χ^2^ = 92.42, df = 2, *p* < 0.001), but not oviposition sequence (χ^2^ = 3.84, df = 2, *p* = 0.147). Complementary Pearson’s chi-square tests confirmed significant associations between molecular detection patterns and developmental time (χ^2^ = 413.79, df = 14, *p* < 0.001; Cramer’s V = 0.57) and host species (χ^2^ = 80.06, df = 2, *p* < 0.001; Cramer’s V = 0.35), whereas the association with oviposition sequence was not significant (χ^2^ = 2.97, df = 2, *p* = 0.227; Cramer’s V = 0.07) (Table 1).

**Figure 1:**
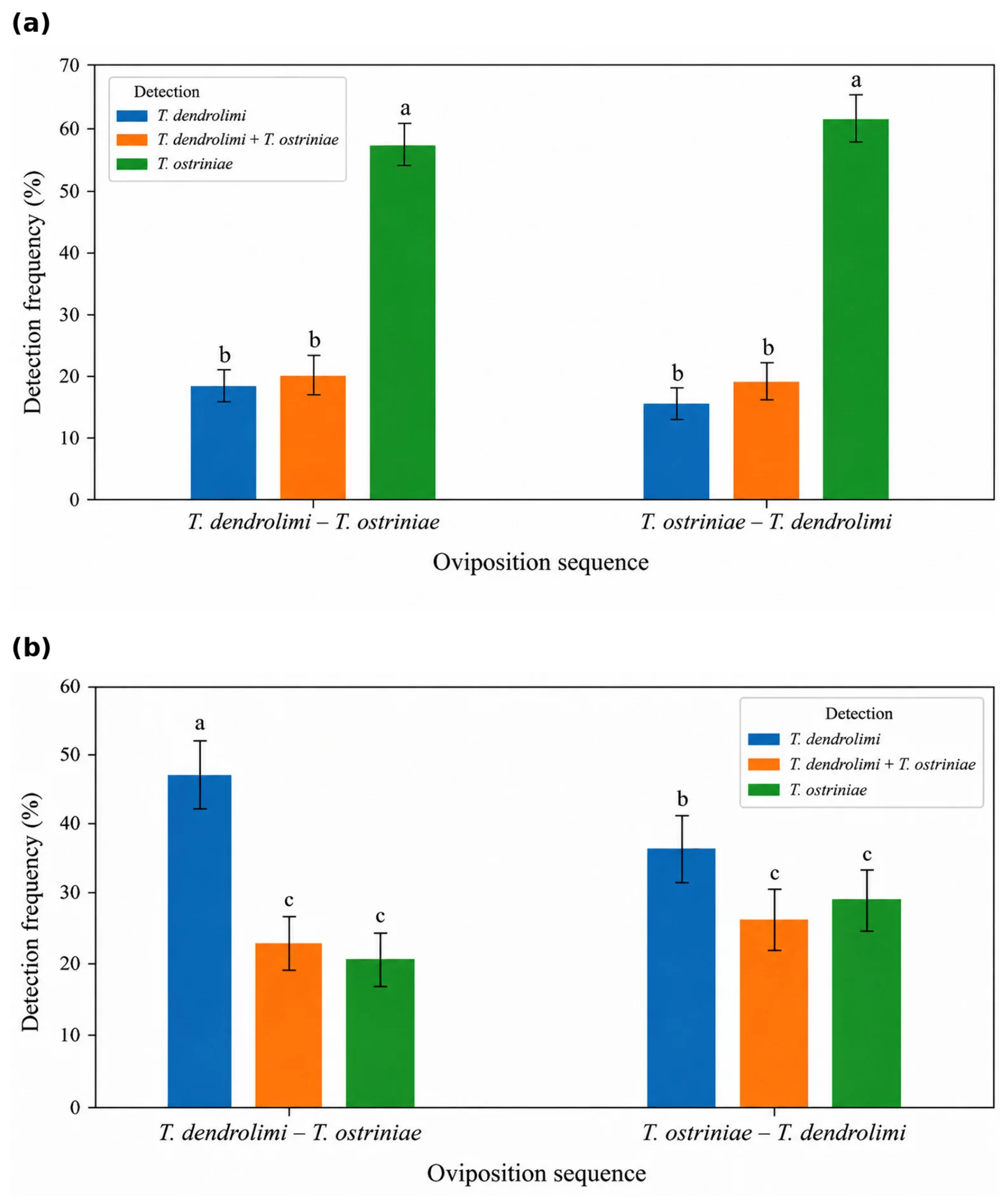
Molecular detection frequencies of *T. dendrolimi*, *T. ostriniae*, and simultaneous detection of both species under different oviposition sequences in (**a**) *O. furnacalis* and (**b**) *C. cephalonica* eggs. Different lowercase letters indicate significant differences among detection categories.

Molecular detection patterns differed between host species but showed no significant overall effect of parasitoid introduction sequence. *T. ostriniae* was the most frequently detected species in *O. furnacalis* eggs under both introduction sequences, whereas *T. dendrolimi* predominated in *C. cephalonica* eggs (Figure 1a,b).

**Figure 2:**
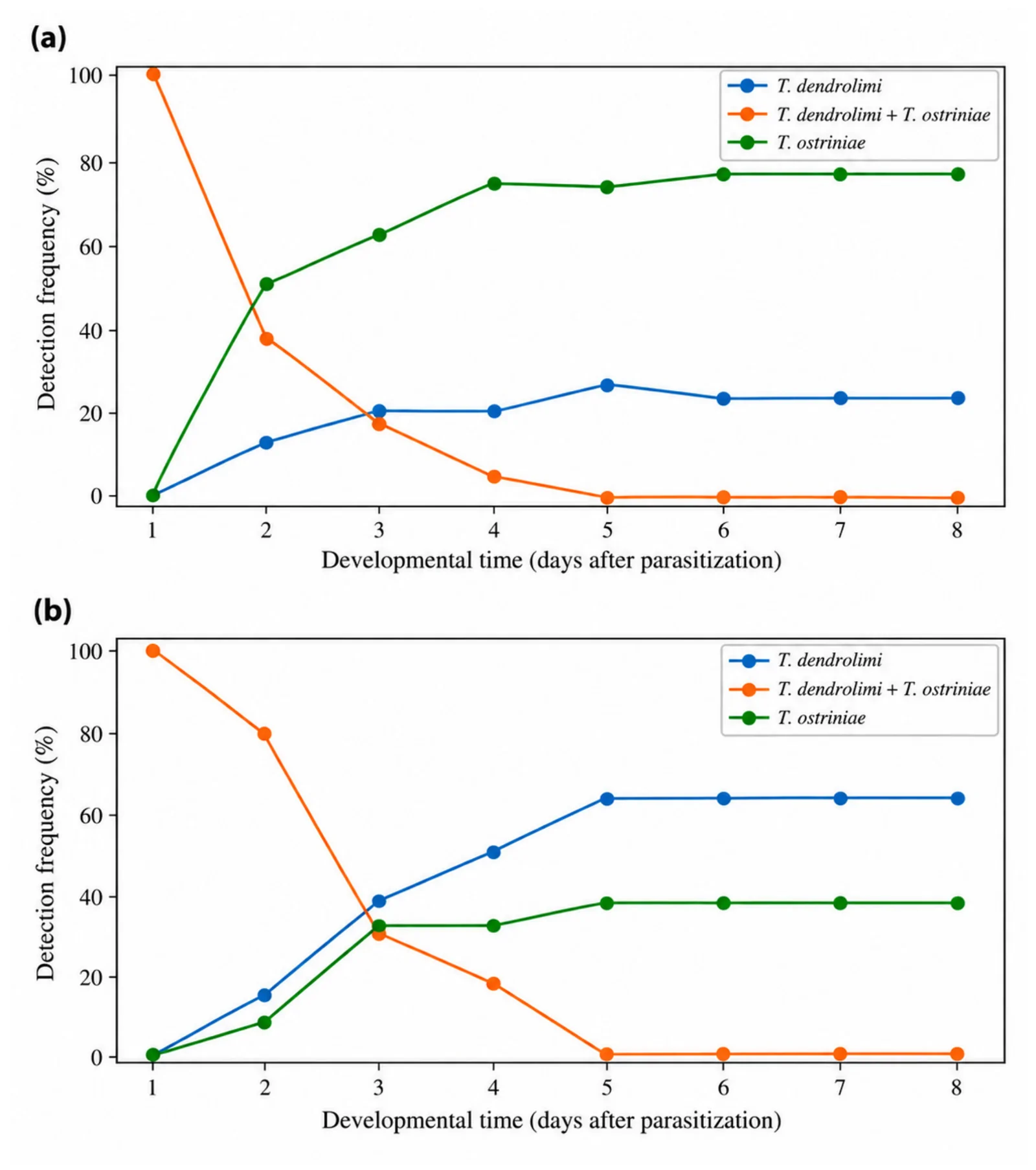
Temporal changes in molecular detection frequencies of *T. dendrolimi*, *T. ostriniae*, and simultaneous detection of both species in (**a**) *O. furnacalis* and (**b**) *C. cephalonica* eggs during days 1–8 after parasitization.

Temporal detection patterns also differed between hosts. In *O. furnacalis* eggs, simultaneous detection declined from 100% on day 1 to approximately 37% on day 2 and became undetectable after day 5. Meanwhile, *T. ostriniae* detection increased to more than 75% during later development, whereas *T. dendrolimi* remained at approximately 20–25% (Figure 2a). In *C. cephalonica* eggs, simultaneous detection similarly declined, while *T. dendrolimi* became predominant, reaching approximately 60% from day 5 onward. *T. ostriniae* detection remained near 38% during later developmental stages (Figure 2b).

## 4. Discussion

### 4.1. Developmental Progression and Effects of Oviposition Sequence

Our results demonstrated that developmental progression was the strongest factor associated with molecular detection patterns between *T. dendrolimi* and *T. ostriniae*. Oviposition sequence showed no significant overall main effect, and inclusion of the two-way interaction terms did not significantly improve model fit. The strong influence of developmental time indicates that competitive interactions are progressively resolved during immature development rather than being determined immediately after host exploitation. These patterns indicate a dynamic transition from early co-detection toward species-specific molecular persistence during parasitoid development within host eggs. Similar patterns have been observed in parasitoid systems, where competitive superiority depends on developmental rate, physiological adaptation, and the ability to exploit host resources during larval development, rather than solely on priority effects associated with the order of host invasion [21,25]. Recent evidence from *Trichogramma* systems further supports this interpretation. Wang et al. [10] investigated competition between *T. ostriniae* and *T. dendrolimi* under different oviposition sequences and host egg densities and demonstrated that competitive outcomes were influenced by parasitoid species identity, host characteristics, and environmental conditions rather than introduction order alone. Therefore, the absence of a significant overall sequence effect suggests that introduction order was not a major determinant of the overall molecular detection patterns under the conditions tested. The predominance of *T. ostriniae* detection in *O. furnacalis* under both introduction sequences, together with the relatively small sequence-related differences observed in *C. cephalonica*, further suggests that introduction order alone did not determine the overall molecular detection patterns. However, some parasitoid systems exhibit strong priority effects, with earlier oviposition providing a competitive advantage [29]. Intrinsic competitive outcomes may also involve physiological mechanisms, including physical and biochemical factors that contribute to the exclusion of competitors [25]. Such differences among studies may arise from variation in host species, time intervals between parasitization events, parasitoid biology, and experimental conditions. In our experiment, the short interval between parasitoid introductions likely minimized the opportunity for the first parasitoid to establish exclusive control of host resources.

### 4.2. Host-Dependent Competitive Superiority

The present study revealed contrasting competitive outcomes between *O. furnacalis* and *C. cephalonica* hosts, with *T. ostriniae* showing greater molecular persistence in *O. furnacalis* eggs and *T. dendrolimi* showing greater persistence in *C. cephalonica* eggs. These host-specific temporal patterns demonstrate that developmental progression was associated with substantial changes in parasitoid molecular detection profiles. Moreover, the contrasting trajectories observed between *O. furnacalis* and *C. cephalonica* indicate that the host environment influenced the relative representation of the two parasitoid species during immature development. The stabilization of species-specific detection patterns during later developmental stages further suggests differential molecular persistence and species-specific representation within the host environment. This finding supports the concept that parasitoid competitive ability is context-dependent and strongly influenced by host quality. Previous research has shown that *T. ostriniae* exhibits superior biological control performance against *O. furnacalis* compared with *T. dendrolimi*, mainly due to higher reproductive success and improved adaptation to *O. furnacalis* eggs [12]. Our molecular evidence extends these observations by demonstrating that this advantage develops during immature stages before adult emergence. Conversely, the increased persistence of *T. dendrolimi* in *C. cephalonica* eggs indicates that host suitability can reverse competitive rankings. Host egg size, nutrient availability, and physiological compatibility are important determinants of parasitoid developmental success [45]. Therefore, a parasitoid species that performs competitively in one host system may not maintain superiority in another host environment. Recent studies also emphasize that competition among parasitoids should be evaluated together with host conditions rather than considering parasitoid species independently. Dong et al. [46] demonstrated that competitive interactions in *T. dendrolimi* were influenced by biological traits and host availability, highlighting the importance of ecological context in determining parasitoid performance. An important consideration is that both parasitoid colonies had been maintained on *C. cephalonica* for multiple generations before experimentation. Although this common rearing regime provided both species with the same immediate laboratory rearing environment, prolonged rearing on a factitious host may induce host-associated conditioning or selection, and species-specific responses to such rearing cannot be excluded. Therefore, the host-dependent molecular persistence patterns observed here should be interpreted within the colony-rearing conditions used in this study. Future reciprocal-rearing experiments, including parallel colonies maintained for several generations on *O. furnacalis* and *C. cephalonica*, would help distinguish intrinsic host-dependent competitive responses from potential effects of rearing history.

### 4.3. Molecular Approaches Reveal Hidden Parasitoid Interactions

Traditional assessments based on emergence rates provide only the final competitive winner and cannot reveal temporary coexistence or developmental displacement inside host eggs. The molecular detection approach used in this study overcame this limitation by identifying *T. dendrolimi*, *T. ostriniae*, and simultaneous *T. dendrolimi* and *T. ostriniae* states throughout development. Recent molecular studies have similarly demonstrated the value of species-specific DNA markers for quantifying parasitoid competition. Zhan et al. [42] developed molecular quantification approaches to compare competitive performance among egg parasitoids and showed that molecular assays provide direct evidence of parasitoid persistence and competitive superiority that may remain undetected through conventional approaches. Thus, our findings support integrating molecular diagnostics into biological control studies because they provide mechanistic information about when and how competitive exclusion occurs.

Future research should investigate the physiological mechanisms responsible for host-dependent dominance between *T. dendrolimi* and *T. ostriniae*. Transcriptomic and proteomic analysis could identify genes associated with host adaptation, immune regulation, and interspecific suppression. In addition, field-scale experiments are required to determine whether laboratory-observed competitive patterns influence biological control efficiency under natural conditions. Combining molecular monitoring with ecological modelling, behavioral studies, and multi-generation experiments will improve the prediction of parasitoid establishment and optimize species selection for integrated pest management programs. Reciprocal-rearing experiments using colonies maintained on different host species would additionally help determine the extent to which rearing history contributes to host-dependent competitive patterns.

## 5. Conclusions

This study demonstrates that competition between *T. dendrolimi* and *T. ostriniae* within host eggs is a time-dependent and host-mediated process. Molecular detection revealed an initial phase of coexistence followed by species-specific persistence patterns during parasitoid development. Developmental time represented the strongest driver of detection transitions, while host species significantly modified competitive outcomes. The greater molecular persistence of *T. ostriniae* in *O. furnacalis* eggs and of *T. dendrolimi* in *C. cephalonica* eggs demonstrates that parasitoid detection patterns differed between the two host environments under the experimental conditions used in this study. Furthermore, oviposition sequence showed no significant overall effect, indicating that introduction order alone did not explain the observed molecular detection patterns under the experimental conditions used in this study. These findings provide new insights into hidden parasitoid interactions occurring before adult emergence and demonstrate the value of molecular approaches for understanding biological control agents. Future studies integrating transcriptomic, physiological, and ecological approaches may further clarify the mechanisms responsible for competitive superiority among parasitoid species. Further reciprocal-rearing experiments will be valuable for determining whether these host-dependent patterns remain consistent across different parasitoid rearing histories. Overall, this study reveals hidden competitive dynamics within host eggs and provides insights for optimizing *Trichogramma* species selection in sustainable biological control programs.

## Figures and Tables

**Table 1:** Pearson’s chi-square tests of associations between experimental factors and molecular detection categories.

Factor	χ^2^ Value	df	*p*-Value	Cramer’s V
Developmental time	413.79	14	<0.001	0.57
Host species	80.06	2	<0.001	0.35
Oviposition sequence	2.97	2	0.227	0.07

## Data Availability

All the data is available in this article.
